# Technical note: Dynamic MRI in a complicated giant posterior urethral diverticulum

**DOI:** 10.4103/0971-3026.73536

**Published:** 2010-11

**Authors:** Prasad R Kundum, Arun K Gupta, Prasad V Thottom, Manisha Jana

**Affiliations:** Department of Radiodiagnosis, All India Institute of Medical Sciences, Ansari Nagar, New Delhi, India

**Keywords:** Dynamic MRI, posterior urethral diverticulum, voiding cystourethrogram

## Abstract

Congenital posterior urethral diverticulum is an uncommon anomaly, sometimes complicated by infection or calculi formation. A conventional voiding cystourethrogram (VCUG) is the most commonly used diagnostic modality. Dynamic magnetic resonance imaging (MRI) has not been frequently described in this entity. We describe a case of posterior urethral diverticulum complicated with secondary calculi, where the patient was evaluated using dynamic MRI and conventional VCUG.

## Introduction

Congenital urethral diverticula in boys are extremely rare, especially those arising from the posterior urethra.[1–3] We report a case of posterior urethral diverticulum containing multiple calculi and with associated genitourinary anomalies. Conventional contrast studies and ultrasound ultrasonography (USG) are usually sufficient for the diagnosis of a urethral diverticulum. We describe a case of a giant posterior urethral diverticulum in a boy, which was diagnosed with the help of dynamic magnetic resonance imaging (MRI).

## Case Report

**Figure 1:**
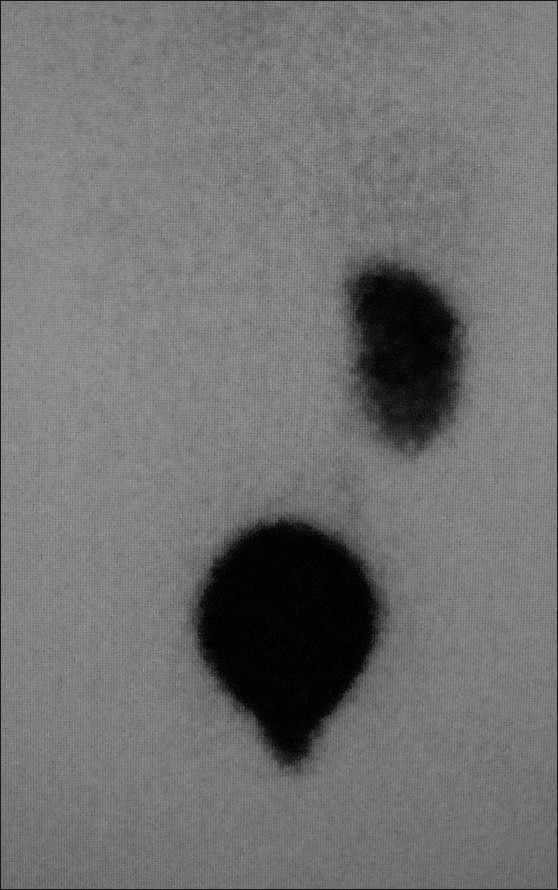
Posterior image from a Tc99m DMSA scintigraphy study reveals absence of the left kidney

An 11-year-old male child presented with complaints of post-void dribbling of urine and pain in the abdomen for the last two years. Physical examination revealed bilateral empty scrotal sacs, with a normally developed phallus. Laboratory evaluation revealed a normal hemogram and normal renal function tests. Microscopic examination of urine showed pus cells, and culture grew *Escherichia coli*. USG revealed a normal right kidney, an empty left renal fossa, and two cystic swellings in the pelvis that were communicating with each other through a narrow channel The posteriorly located cystic swelling contained echogenic contents, with acoustic shadowing suggestive of calculi. The left kidney could not be visualized. We suspected a urinary bladder diverticulum or a midline cystic lesion such as a Mullerian duct/prostatic utricular cyst. The absent left kidney was confirmed with a renal Tc99m dimercaptosuccinic acid (DMSA) nuclear scan [Figure 1].

**Figure 2:**
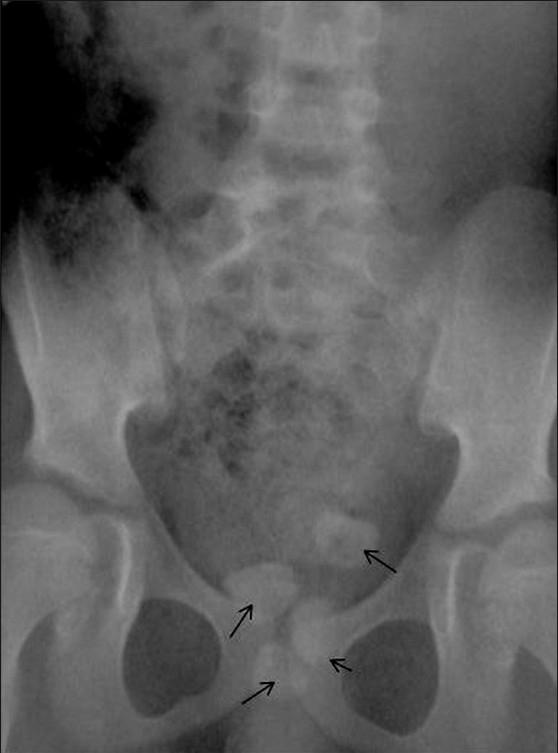
Anteroposterior radiograph of the abdomen and pelvis reveals multiple radiodense calculi (arrows) in the pelvis, overlying the symphysis pubis

**Figure 3:**
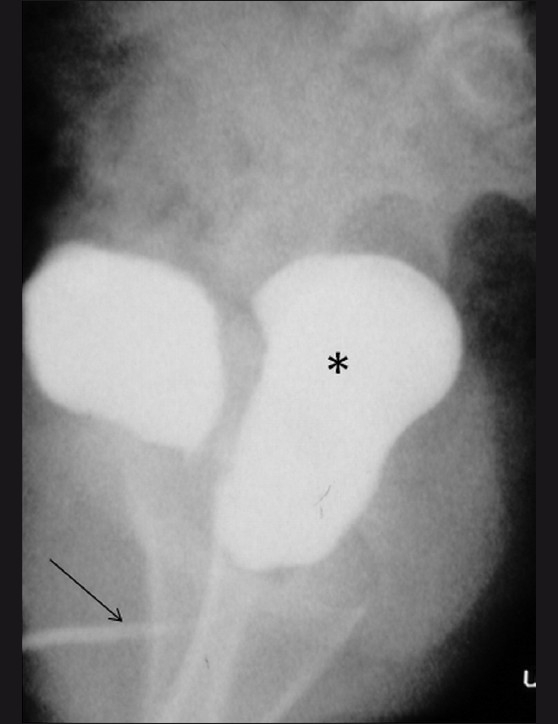
Lateral voiding cystourethrogram (VCUG) reveals a contrastfilled structure (*) posterior to the bladder, contiguous with the anterior urethra (arrow). The posterior urethra cannot be identified separately

Subsequently, a voiding cystourethrogram (VCUG) was performed. The plain radiograph revealed multiple calculi in the pelvis, overlying the symphysis pubis [Figure 2]. The VCUG showed two contrast-filled cystic structures in the pelvis that were communicating with each other through a narrow channel [Figure 3]. The posteriorly located cystic structure contained multiple calculi and the urethra was seen to descend from it. We considered the possibilities of a posterior urethral diverticulum or a narrow-necked urinary bladder diverticulum with multiple bladder calculi.

**Figure 4:**
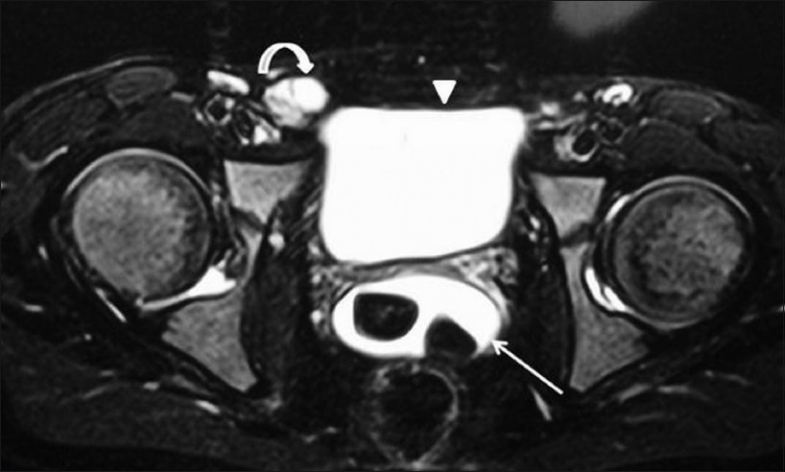
Fat-saturated, T2W, TSE axial image of the pelvis shows the bladder anteriorly with another fluid-filled structure, containing multiple hypointense calculi (arrow), posterior to the bladder (arrowhead). The right testis is seen as a hyperintense oval structure in the right inguinal canal (curved arrow)

**Figure 5:**
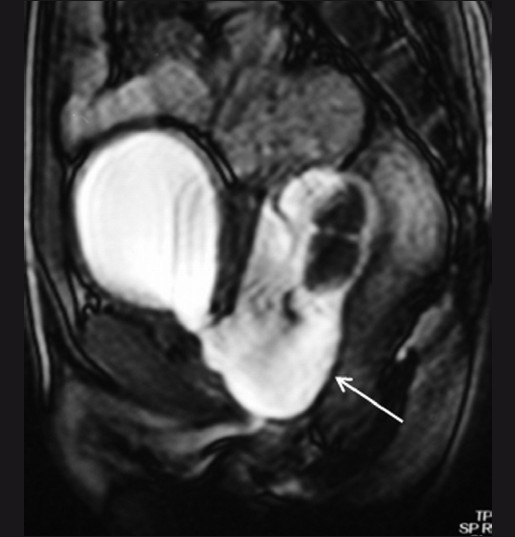
Sagittal dynamic gradient-echo image (TruFISP) in the resting phase reveals a large fluid-containing structure (arrow) posterior to the urinary bladder; it contains multiple calculi. The structure is contiguous with the posterior urethra, suggesting the possibility of a posterior urethral diverticulum

**Figure 6:**
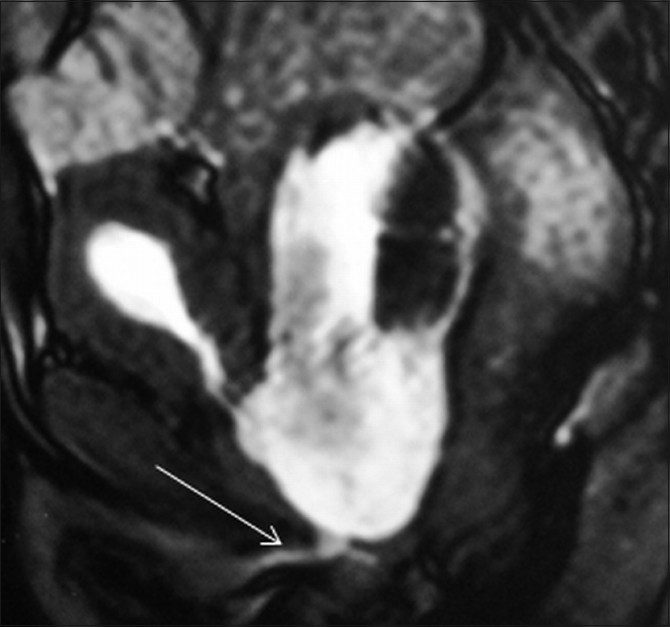
Sagittal dynamic gradient-echo image (TruFISP) in the voiding phase shows emptying of the bladder and filling of the diverticulum. The anterior urethra is well seen (arrow). Note the absence of prostatic parenchyma

**Figure 7:**
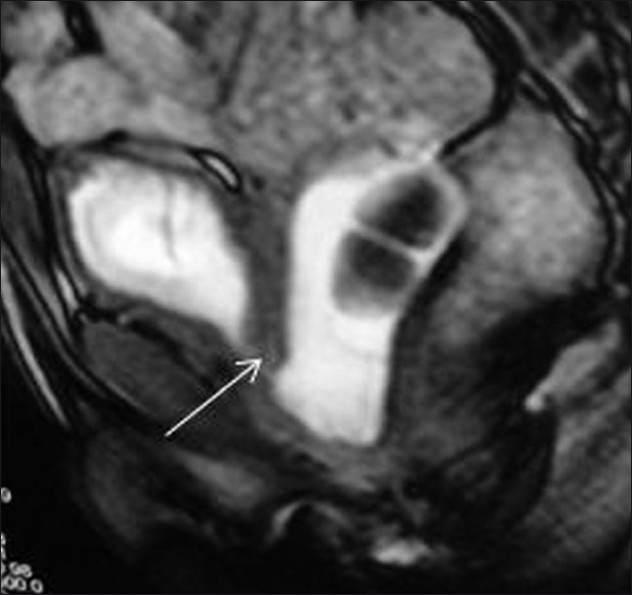
Sagittal dynamic gradient-echo image (TruFISP) after the end of voiding shows retrograde filling of the urinary bladder from the diverticulum through an incompetent internal urethral sphincter (arrow)

MRI was then performed on a 1.5-T system (Siemens Sonata, Erlangen, Germany) for delineation of the anatomy and the associated anomalies. Routine T1W (TR 709 ms, TE 13 ms) and T2W images with fat suppression (TR 5870 ms, TE 101 ms) were acquired using a 4-mm slice thickness and a 256 × 256 matrix. The T2W axial image of the pelvis revealed a fluid-filled structure posterior to the bladder [Figure 4]. The right testis was seen in the inguinal canal [Figure 4]. Dynamic MRI was performed in the sagittal plane using a steady-state free-precession gradient-echo sequence (True FISP) (TR 4.3 ms, TE 2.1 ms matrix 256 × 256, flip angle 12°) in multiple phases of micturition. During the resting phase the urinary bladder appeared as a structure filled with urine and the diverticulum showed fluid signal intensity with areas of signal void (suggestive of calculi) [Figure 5]. During the voiding phase, the bladder musculature was seen to contract, with resultant gradual emptying of the bladder (seen as reducing bladder volume) and filling of the diverticulum with urine [Figure 6]. At the end of voiding, the bladder had emptied completely but urine was retained in the diverticular sac, which resulted in subsequent retrograde filling of the urinary bladder through an incompetent bladder neck sphincter [Figure 7]. The left kidney, prostate, and seminal vesicles were absent.

Cystourethroscopy revealed the opening (neck of the diverticulum) in the prostatic urethra. A surgical resection through a perineal approach was performed, and histopathology confirmed the presence of transitional epithelium in the diverticulum.

## Discussion

A urethral diverticulum is a sac-like outpouching of the urethral mucosa or simply a tubular sac connecting into the urethra at any point in its course. It is more common in females than males.[1–3] These diverticula are classified as true (congenital) and false (acquired) types.

Unlike anterior urethral diverticula, the majority of posterior urethral diverticula are acquired in origin.[1] Patients with a posterior urethral diverticulum may be asymptomatic or may have variable symptoms such as difficulty in urination, urgency, lower abdominal swelling, perineal discomfort, and backache.[4–6] These cases can be further complicated by infection and secondary calculi formation (more commonly observed in females) as in our case.[5]

USG and VCUG are usually the initial modalities of choice and are adequate in most cases. However, a giant posterior urethral diverticulum may cause diagnostic difficulties due to distorted anatomy, and additional imaging may be required for proper preoperative assessment.

MRI, because of its good soft tissue contrast resolution, allows superior visualization of the urethra and the adjacent structures. It is also possible to visualize the diverticulum sac which may not be opacified on contrast studies.[7] Dynamic MRI with fast gradient-echo sequences, combines the advantage of superior soft tissue contrast and a reasonable temporal resolution, thus allowing excellent visualization of the lower urinary tract, both in the resting as well as the voiding phases.[8] Dynamic MRI of the urethra has also been earlier used in pelvic floor dysfunction and urethral hypermobility disorders.[7,8] Transrectal USG can be used in older individuals for screening suspected cases of posterior urethral diverticulum.[9] Although transrectal USG provides good spatial resolution, it has the drawback of being operator dependent. High-resolution dynamic MRI can provide good anatomic detail and superior soft tissue contrast resolution.

In conclusion, the excellent soft tissue contrast, the lack of exposure to radiation, and the good temporal resolution that the newer generation fast gradient-echo sequences provide make dynamic imaging possible. MRI can be the ideal imaging modality for evaluation of the genitourinary tract in children.[7,8] Dynamic MRI can be used in older children as a problem-solving tool in complicated urinary tract anatomy and multiple-associated anomalies, where it can provide the operating surgeon with adequate anatomical and functional information.
